# Association of socioeconomic and behavioral factors with adult mortality: analysis of data from verbal autopsy in Addis Ababa, Ethiopia

**DOI:** 10.1186/1471-2458-13-634

**Published:** 2013-07-08

**Authors:** Awoke Misganaw, Damen Haile Mariam, Tekebash Araya

**Affiliations:** 1 Addis Ababa Mortality Surveillance Program, College of Health Sciences, Addis Ababa University, Addis Ababa, Ethiopia; 2 School of Public Health, College of Health Sciences, Addis Ababa University, Addis Ababa, Ethiopia

**Keywords:** Verbal autopsy, Socioeconomic factors, Tobacco, Alcohol, Khat, Adult deaths, Causes of mortality, Ethiopia

## Abstract

**Background:**

Changes in socioeconomic status, lifestyle and behavioral factors among the urban population in Ethiopia is resulting in a shift in the causes of mortality.

We used verbal autopsy data from 2006 to 2009 to measure the association of socioeconomic and behavioral factors with causes of mortality in Addis Ababa, Ethiopia.

**Methods:**

A total of 49,309 deaths from burial surveillance were eligible for verbal autopsy for the years 2006 to 2009. Among these, 10% (4,931) were drawn randomly for verbal autopsy of which 91% (4,494) were adults of age ≥15 years. Verbal autopsies, used to identify causes of death and frequency of risk factors, were completed for 3,709 (83%) of the drawn sample.

**Results:**

According to the results of the verbal autopsy, non-communicable diseases caused 1,915 (51%) of the total adult deaths, while communicable diseases and injuries caused 1,566 (42%) and 233 (6%) of the deaths respectively.

Overall, frequent alcohol (12%) and tobacco consumption (7%) were highly prevalent among the deceased individuals; both because of communicable diseases (HIV/AIDS and tuberculosis) as well as due to non-communicable diseases (malignancy, cardiovascular and chronic liver diseases). HIV/AIDS (AOR = 2.14, 95% CI [1.52-3.00], p < 0.001) and chronic liver diseases (AOR = 3.09, 95% CI [1.95-4.89], p < 0.001) were significantly associated with frequent alcohol consumption, while tuberculosis was associated with both frequent alcohol (AOR = 1.61, 95% CI [1.15-2.24], p = 0.005) and tobacco consumption (AOR = 1.67, 95% CI [1.13-2.47], p < 0.010). Having low educational status, being female and being within the age range of 25 to 44 years were positively associated with HIV/AIDS related mortality. Individuals aged 45 years and above were 3 to 6 times more likely to have died due to cardiovascular diseases compared with those within the 15 to 24 years age group.

**Conclusion:**

The findings from the analysis suggest that public health interventions targeting HIV/AIDS, tuberculosis, as well as non-communicable diseases need to consider behavioral factors related to alcohol, tobacco and *khat* consumption. We also recommend large scale national level studies to further assess the specific contributions of these risk factors to the burden of mortality in the country.

## Background

Changes in socioeconomic, behavioral and lifestyle factors among the population in developing countries have contributed to the increasing prevalence of non-communicable diseases, superimposed on the existing challenges of communicable diseases [1]. The major non-communicable conditions such as cardiovascular diseases, malignancy and diabetes are strongly associated and causally linked with over-consumption of tobacco and alcohol at global level, with 9% of non-communicable diseases and 71% of lung cancer deaths worldwide being attributed to tobacco alone [2]. The basic causes for these risk factors can also be traced to socio-economic factors such as education and occupational status. Therefore, modifying these basic causes is more likely to have amplified effects, by influencing multiple proximal factors with the potential yield fundamental and sustained improvements in population health [3].

Another social custom that has been implicated as a risk factor for non-communicable diseases is chewing *khat* (a plant native to the horn of Africa and the Arabian Peninsula) [4-6]. Fresh leaves of *khat* is chewed with its twigs for its pleasurable effects, and its frequent consumption is associated with a number of undesirable outcomes [7][8]. In areas where large amounts of *khat* are consumed frequently, such as Yemen, significant and independent association has been reported with risk for acute myocardial infarction, in a strong dose–response manner [9]. The leaves of the *khat* plant, which contain amphetamine-like compounds (cathinone and cathine), are implicated as having undesired effects on blood pressure and heart rate [8]. Cathinone increases blood pressure and heart rate through an amphetamine-like release of nor-adrenaline (nor-epinephrine) from peripheral neurons [10-12].

Recent evidence indicates the prevalence of double burden in mortality in urban areas of Ethiopia, caused by both non-communicable (52%) and communicable (48%) diseases [13]. This indicates the need for further explaining the root socio-economic and behavioral factors that caused such epidemiologic transition in order to properly inform health policy and decision making. Therefore, the overall aim of this study was to measure the association of socioeconomic and behavioral factors with various causes of death in the urban setting of Ethiopia.

## Methods

This study is part of the Addis Ababa Mortality Surveillance Program (AAMSP) that uses the verbal autopsy method on adult mortality. As usable mortality data are lacking in Ethiopia to measure the impact of socioeconomic and behavioral factors on causes of mortality, it has been necessary to apply the verbal autopsy method on data from burial surveillance for such analysis. The AAMSP analyzes data from surveillance of burials in Addis Ababa to capture causes of adult deaths. The burial surveillance has been conducted since 2001 in all cemeteries under the city limit of Addis Ababa. Addis Ababa harbors 89 cemeteries (670 are church based, 9 are mosque based, while 10 are municipality owned cemeteries) [14]. The data used are for the period of September, 2006 to December, 2009.

The burial surveillance is used as a sampling frame for the verbal autopsy procedure [15]. As cremation is not practiced in Addis Ababa, all burials of deaths are conducted at the above mentioned religious or municipality based cemeteries. Thus, in principle, the burial surveillance captures all deceased residents of Addis Ababa, although biases exist because residents may die and/or be buried outside the capital just as non-residents may be buried inside Addis Ababa. Some of these biases are mostly identified and corrected while others inevitably go unnoticed.

The burials are registered by cemetery clerks who are well trained and regularly given annual refresher trainings. They report all deaths (n ≈ 18,000 per year) from cemeteries using structured forms. They collect the information from relatives or close friends during burial ceremonies. The information include: date of burial, age, name, sex, address, marital status, region of birth, ethnicity, religion, and a lay reported cause of death [15].

Adult deaths aged 15 years and above that were captured by the burial surveillance in 2007 were 18,013, making age specific death rate of 8.9 per 1000 for Addis Ababa. This figure is very close to the results of the census by the Ethiopian Statistical Authority for the same year (18,686 total deaths of aged 15 years with age specific death rate of 9.2 per 1000) [16]. Adult deaths captured by the burial surveillance in 2008 and 2009 were 17,984 and 18,154 respectively. Overall, the burial surveillance has identified 58,010 deaths during September 2006 to December 2009. Among these, 49,309 (85%) deaths were eligible for verbal autopsy procedures while the remaining could not be subject to such procedures because the burials were conducted without close relatives or friends who could provide information for the verbal autopsy interviews.

### Verbal autopsy

Verbal Autopsy is interviewing the relatives or caregivers about the signs, symptoms, lifestyle behaviors and other characteristics experienced by the deceased before their death and the circumstances surrounding their death [17]. The verbal autopsy questionnaire was piloted and adapted to local situation from a standardized WHO and International Network of field sites with continuous Demographic Evaluation of Populations and their Health in developing countries (INDEPTH Network) verbal autopsy questionnaires [17,18]. Some modifications were made on the questionnaire for the purpose of enhancing local comprehension and ensuring cultural acceptability. This helped the program to obtain internationally comparable verbal autopsy data with the burial surveillance approach. The questionnaire consists of identification of respondents and care givers, identification of the deceased, death related information, signs and symptoms during illness and list of possible risk factors.

Three data collectors, who are high school graduates with similar previous experiences, are deployed to visit each of the sampled households on average of two months after the death occurred. Data collectors are given extensive training on the objective of the program, on the questionnaire and on skills of interview. To maintain the quality of the data, annual refresher trainings, monthly supervisions and weekly meetings are conducted.

After data entry and cleaning, 10% (4,931) of the deaths were randomly drawn using the Visual Basic Computer Program for verbal autopsy. Among the drawn sample, 91% (4,494) were adults of age 15 years and above, and 9% (437) were under 15 years of age. Verbal autopsies were completed among 3,709 (83%) of the adult deaths, while care givers were not willing to participate or not available with repeated visits for the rest of the cases.

All completed verbal autopsies also underwent physician review before assigning underlying causes of death. Initially, two physicians reviewed every completed verbal autopsy interview blindly to assign possible cause/s of death. Any inconsistent case within the initial review is referred to a third physician. If the results of the three physicians are inconsistent, it would be referred to a panel discussion. In the few cases where it is difficult to arrive at a probable cause of death even after panel discussion, the cause of death will be labeled as “undetermined”. Finally, the International Classification of Diseases Version 10 (ICD-10) is used for standardization and for comparison with other studies.

### Behavioral risk factors

The adult verbal autopsy questionnaire for this study contains similar questions to that of the internationally standard verbal autopsy questionnaire on consumption of tobacco and alcohol [17]. In addition, *khat* chewing has been included as a locally important behavioral risk factors [19] with the globally identified risk factors of tobacco and alcohol consumption [1]. Since a system for surveillance of behavioral risk factors is lacking in Ethiopia, the verbal autopsy with burial surveillance can be considered as one alternative approach to examine the prevalence of behavioral risk factors and their associations with mortality. The questionnaire elicited information on the duration and frequency of tobacco and alcohol consumption as well as *khat* chewing. Alcohol consumption and *khat* chewing were categorized into: frequently in a week (at least four times a week) and occasionally; while tobacco use was categorized as frequently (at least once per day) and occasionally. Furthermore, there were categories for getting drunk with alcohol as frequently, once in a week and occasionally.

### Demographic and socioeconomic factors

The causes of deaths were analyzed in relation to demographic (age, sex and marital status) and socioeconomic (religion, educational and occupation) factors. The age of the deceased (in completed years) is categorized as: 15 to 24, 25 to 44, 45 to 54, 55 to 64, and 75 and older. Educational status is categorized as: no education, primary education, and secondary education, above secondary and others (traditional). Occupation is categorized as: professional (technical/managerial/sales/clerical/self-employed), manual labor (skilled/unskilled), housewives, unemployed, retired, and others (students, farmers).

### Data management and analysis

Double data entry has been implemented with Microsoft Access program followed by a thorough data cleaning using STATA driven .do files. For this analysis the age group 15 years and above was selected due to the fact that this group is economically productive and is highly affected by over-consumption of the substances at issue [20]. Using this age group for analysis is also considered appropriate since the burial surveillance method is prone to under-reporting child deaths [14,15,21].

Further analysis focused on identified leading causes of diseases in the study area such as cardiovascular diseases, malignancy, chronic liver diseases, HIV/AIDS and tuberculosis and presented meaningful findings [13]. The 2006 Global Burden of Diseases classification was adapted to classify causes of death in this study. The classification categorized diseases into: Group I (communicable diseases, maternal conditions and nutritional deficiencies); Group II (non-communicable causes); and Group III (injuries) [22].

Statistics such as frequencies and cross tabulations are applied to examine the distribution of each demographic, socio economic and behavioural risk factors. To compare proportions of behavioural risk factors by causes of death, the chi-squared test is used. Further, binary logistic regression was applied to examine the relationship between demographic, socioeconomic and behavioural risk factors with selected causes of death using the STATA software (Stata Corp LP, College Station, Texas).

### Ethical considerations

The protocol for the Addis Ababa Mortality Surveillance Program has been approved by the Institutional Review Board (IRB) of the College of Health Sciences, Addis Ababa University as well as the Ethics Committee of the Ethiopian Ministry of Science and Technology. Permission for conducting the study within the burial sites has also been obtained from the local authorities. Individual interviews are conducted after getting verbal informed consent from the immediate kin and caregivers of the deceased. At the program office, individual information is accessible only to the research team and is kept strictly confidential.

## Results

Among the total of the deceased adults on whom the verbal autopsies were completed, 50% were females and 60% were within the age range of 15 to 64 years. The median age of the deceased was 55 years (ranging 15–105). Regarding educational status, 44% had completed elementary and/or secondary school while 38% were illiterates. Thirty nine percent were engaged in some income generating activity through employment or self employment. Thirty percent were housewives, 17% were retired and 88% were Orthodox Christians by religion (Table 1). Based on the Global Burden of Diseases classification, 1,915 (51%) of the deaths were due to non-communicable diseases, while 1,566 (42%) and 233 (6%) were due to communicable diseases and injuries respectively. The rest 254 cases were undetermined.

**Table 1:** Socio-demographic characteristic of deceased adults (N = 3709) in Addis Ababa, Ethiopia, 2006 – 2009

**Socioeconomic characteristics**	**Males n (%)**	**Females n (%)**
**Age**		
15-24	94 (5)	123 (7)
25-34	261 (14)	270 (15)
35-44	286 (15)	241 (13)
45-54	250 (13)	237 (13)
55-64	229 (12)	234 (13)
65-74	337 (18)	262 (14)
75-84	248 (13)	221 (12)
85^+^	159 (9)	257 (14)
**Religion**		
Orthodox	1649 (86)	1629 (88)
Muslim	170 (9)	166 (9)
Others	45 (2)	50 (3)
**Ethnicity**		
Amhara	933 (50)	1001 (54)
Oromo	456 (25)	453 (25)
Guraghe	248 (13)	212 (12)
Others	227 (12)	179 (10)
**Education status**		
No education	484 (26)	971 (53)
Elementary School	486 (26)	405 (22)
Secondary School	465 (25)	276 (15)
College/University	222 (12)	74 (4)
Others (church education)	207 (11)	119 (6)
**Occupation**		
Manual labor (skilled/unskilled)	319 (17)	216 (12)
Employed (professional, technical, managerial, self-employed, sales and services)	705 (38)	248 (13)
Unemployed	200 (11)	94 (5)
Housewives	-	1090 (59)
Retired	465 (25)	79 (4)
Others (Students, farmers)	52 (3)	46 (3)
Unknown	123 (7)	72 (4)
**Marital status**		
Single	515 (28)	289 (16)
Married	1032 (55)	446 (24)
Separated	48 (3)	133 (7)
divorce	58 (3)	139 (8)
Widowed	211 (11)	838 (45)

### Prevalence of alcohol, tobacco and *khat* consumption among the deceased adults

Among all the deceased adults, a significant proportion (29%) had ever used alcohol (47% of the males and 10% of the females). Nearly 12% were frequent consumers of alcohol (22% of the males and 2% of the females). In addition, 10% of the deceased adults had ever smoked tobacco, 7% (all males) being frequent smokers. Regarding the duration of smoking, 6% had smoked for more than 11 years. Approximately 8% of the deceased adults had ever chewed *khat*, 4% them being frequent chewers (Table 2). In summary, alcohol was the substance most frequently consumed, followed by tobacco then *khat.* Males were more frequent consumers of all the three substances.

**Table 2:** **Tobacco, alcohol and *khat *use by sex among deceased Adults in Addis Ababa, Ethiopia, 2006-2009**

**Health risk behaviors**	**Males (n = 1864)**	**Females (n = 1845)**	**Total (n = 3709)**
	**n (%)**	**n (%)**	**n (%)**
**Alcohol use**			
Ever alcohol use	869 (47)*	188 (10)	1055 (29)
Frequently alcohol use	411 (22)*	43 (2)	454 (12)
Occasionally alcohol use	424 (23)*	131(7)	555 (15)
**Getting drunk of alcohol use**			
Frequently get drunk	247 (13)*	22 (1)	269 (7)
Occasionally get drunk	187 (10)*	21 (1)	208 (6)
**Tobacco use**			
Ever tobacco use	337 (18)*	15 (1)	352 (10)
Frequently tobacco use	244 (13)*	8 (0.4)	252 (7)
Occasionally tobacco use	29 (2)	-	29 (1)
**Duration of tobacco use**			
Tobacco used, ≤ 5years	29 (2)	-	29 (1)
Tobacco used ,5 to10 years	26 (1)*	2 (0.1)	28 (1)
Tobacco used, 11 and above years	228 (12)*	9 (1)	237 (6)
Tobacco use till sick	217 (12)*	10 (1)	227 (6)
Stopped tobacco use before being sick	100 (5)*	2 (0.1)	102 (3)
***Khat *Use**			
Ever *khat* use	238 (13)*	39 (2)	277 (8)
Frequently *khat* use	145 (8)*	14 (1)	159 (4)
Occasionally *khat* use	77 (4)*	19 (1)	96 (3)

Note: * P < 0.001. P value computed by comparing males and females using chi- squared test.

### Patterns of alcohol, tobacco and *khat* consumption in relation to causes of death

About 14% of the deceased adults whose causes of death were assigned to communicable diseases were reported have been frequent alcohol consumers. More specifically, 18% of those whose causes of death were assigned to be due to tuberculosis and 16% of those whose causes of death were assigned to HIV/AIDS were reported to be frequent alcohol consumers. On the other hand, among those whose causes of death were assigned to non-communicable diseases, 11% were reported to be alcohol consumers. More specifically, highest frequency of alcohol consumption was evident among those that died from poisoning (43%), chronic liver diseases (27%), cardiovascular diseases (9%), and malignancies (6%) (Table 3).

**Table 3:** Alcohol use among deceased adults in relation to causes of death (N = 3709), Addis Ababa, Ethiopia, 2006-2009

**Causes of death**	**Number**	**Frequent alcohol use**	**Occasional alcohol use**	No alcohol use**
	**n**	**n(%)**	**n(%)**	**n(%)**
**I. Communicable diseases**	1566	225 (14)*	241 (16)	1100 (70)
HIV/AIDS	707	111 (16)*	90 (13)	506 (72)
Tuberculosis	460	82 (18)*	79 (17)	299 (65)
Respiratory infections	102	10 (10)	16 (16)	76 (75)
Diarrheal disease	115	15 (13)	21 (18)	79 (69)
Meningitis	47	2 (4)	6 (13)	39 (83)
**II. Non communicable disease**	1915	211 (11)*	287 (15)	1417 (74)
A. Malignant neoplasm	386	24 (6)*	43 (11)	319 (83)
Stomach Cancer	73	6 (8)	10 (14)	57 (78)
Breast Cancer	43	1 (2)*	1 (2)	41 (96)
Uterine Cancer	47	1 (2)*	1 (2)	45 (96)
Liver Cancer	37	4 (11)	4 (11)	29 (78)
Cancer of cervix	19	1 (5)	-	18 (95)
Colon and rectal cancer	27	2 (7)	5 (16)	20 (74)
Other neoplasm	145	12 (8)	20 (14)	113 (78)
B. Diabetes mellitus	183	18 (10)	29 (16)	136 (74)
C. Neuropsychiatry	76	11 (15)	15 (20)	50 (66)
D. Cardiovascular diseases	885	81 (9)*	141 (16)	663 (75)
Hypertensive disease	426	35 (8)*	71 (17)	320 (75)
Stroke	404	40 (10)	72 (18)	292 (72)
Congestive heart failure	239	17 (7)*	34 (14)	188 (79)
Myocardial infarction	84	8 (10)	14 (17)	62 (74)
E. Respiratory diseases	93	8 (9)	12 (13)	73 (79)
Asthma	73	7 (10)	10 (14)	56 (77)
F. Digestive diseases	328	61 (19)*	55 (17)	212 (65)
Chronic liver disease	165	45 (27)*	28 (17)	92 (56)
Peptic ulcer disease	31	2 (7)	5 (16)	24 (77)
G. Genitourinary disease	137	12 (9)	24 (18)	101 (74)
Chronic renal failure	112	10 (9)	19 (17)	83 (74)
**III. Injuries**	233	22 (9)	37 (16)	174 (75)
A. Unintentional	102	4 (4)*	11 (11)	87 (85)
Road traffic accidents	83	3 (4)*	8 (10)	72 (87)
B. Intentional (Suicide…)	79	7 (9)	12 (15)	60 (76)
Poisoning	28	12 (43)*	4 (14)	12 (43)
Undetermined	254	22 (9)	34 (13)	198 (78)

Note: * P < 0.03. P value obtained by comparison of frequent alcohol use, occasional alcohol use and non alcohol use by chi- squared trend test.

** non alcohol use is a reference category.

Regarding tobacco, those whose causes of death were assigned to communicable diseases (12% of the deaths assigned to tuberculosis and 10% of the deaths assigned to HIV/AIDS) were reported to have been frequent tobacco smokers. On the other hand, 6% of those who died due to non-communicable diseases (5% of those due to cardiovascular diseases and 36% of those due to poisoning) were reported to have been frequent tobacco smokers. Frequency of tobacco smoking was also greater among those that died of chronic liver diseases (Table 4).

**Table 4:** Tobacco use among deceased adults in relation to causes of death (N = 3709), Addis Ababa, Ethiopia, 2006-2009

**Cause of death**	**Number**	**Frequent tobacco use**	**Occasional tobacco use**	No tobacco use **
	**n**	**n(%)**	**n(%)**	**n(%)**
**I. Communicable diseases**	1566	135 (9)*	15 (1)	1416 (90)
HIV/AIDS	707	69 (10)*	7 (1)	631 (89)
Tuberculosis	460	57 (12)*	8 (2)	395 (86)
Respiratory infections	102	1 (1)*	2 (2)	99 (97)
Diarrheal disease	115	6 (5)	1 (1)	108 (94)
Meningitis	47	2 (4.3)	-	45 (96)
**II. Non communicable disease**	1915	108 (6)*	11 (1)	1796 (94)
A. Malignant neoplasm	386	15 (4)*	1 (0.3)	370 (96)
Stomach cancer	73	4 (6)	-	69 (95)
Breast cancer	43	-	-	43 (100)
Uterine cancer	47	-	-	47 (100)
Liver cancer	37	1 (3)	-	36 (97)
Cancer of cervix	19	-	-	19 (100)
Colon and rectal cancer	27	2 (7)	-	25 (92)
Other neoplasm	145	8 (6)	1 (1)	136 (94)
B. Diabetes mellitus	183	12 (7)	2 (1)	169 (92)
C. Neuropsychiatry	76	5 (7)	2 (3)	69 (91)
D. Cardiovascular diseases	885	44 (5)*	3 (0.3)	838 (95)
Hypertensive disease	426	17 (4)*	2 (1)	407(96)
Stroke	404	18 (5)	2 (1)	384 (95)
Congestive heart failure	239	13 (5)	1 (0.4)	225 (94)
Myocardial infarction	84	10 (12)	-	74 (88)
E. Respiratory diseases	93	-	-	93 (100)
Asthma	73	-	-	73 (100)
F. Digestive diseases	328	29 (9)	3 (1)	296 (90)
Chronic liver disease	165	17 (10)	2 (1)	146 (89)
Peptic ulcer disease	31	1 (3)	1 (3)	29 (94)
G. Genitourinary disease	137	6 (4)	-	131 (96)
Chronic renal failure	112	5 (4.5)	-	107 (96)
**II. Injuries**	233	19 (8)	3 (1)	211 (91)
A. Unintentional	102	6 (6)	1 (1)	95 (93)
Road traffic accidents	83	4 (5)	1 (1)	78 (94)
B. Intentional (Suicide…)	79	9 (11)	-	70 (89)
Poisoning	28	10 (36)*	-	18 (64)
Undetermined	254	7 (3)*	1(0.4)	246 (97)

Note: * P < 0.03. P value obtained by comparison of frequent tobacco use, occasional tobacco use and non tobacco use by chi- squared trend test.

**No tobacco use is a reference category.

*Khat* chewing was reported among 6% of those whose causes of death were assigned to communicable diseases (9% of those deaths assigned to HIV/AIDS and 7% of those deaths assigned to tuberculosis); while it was also reported among 10% of those whose causes of death were assigned to intentional injuries. Frequency of *khat* chewing was also greater among those that died of digestive diseases (Table 5).

**Table 5:** ***Khat *use among deceased adults in relation to causes of death (N = 3709), Addis Ababa, Ethiopia, 2006-2009**

**Cause of death**	**Number**	**Frequent *khat *use**	**Occasional *khat *use**	No *khat *use **
	**n**	**n (%)**	**n (%)**	**n (%)**
**I. Communicable diseases**	1566	93 (6)*	46 (3)	1427 (91)
HIV/AIDS	707	60 (9)*	29 (4)	618 (87)
Tuberculosis	460	30 (7)*	13 (3)	417 (91)
Respiratory infections	102	2 (2)	-	100 (98)
Diarrheal disease	115	4 (4)	3 (3)	108 (94)
Meningitis	47	1 (2)	1 (2)	45 (96)
**II. Non Communicable disease**	1915	64 (3)*	37(2)	1814 (95)
A. Malignant neoplasm	386	7 (2)*	9 (2)	370 (96)
Stomach cancer	73	2 (3)	1 (1)	70 (96)
Breast cancer	43	-	1 (2)	42 (98)
Uterine cancer	47	-	1 (2)	46 (98)
Liver cancer	37	-	-	37 (100)
Cancer of cervix	19	-	-	19 (100)
Colon and rectal cancer	27	1 (4)	-	26 (96)
Other neoplasm	145	3 (2)	4 (3)	138 (95)
B. Diabetes mellitus	183	6 (3)	3 (2)	174 (95)
C. Neuropsychiatry	76	4 (5)	3 (4)	69 (91)
D. Cardiovascular diseases	885	25 (3)*	13 (2)	847 (96)
Hypertensive heart disease	426	9 (2)*	5 (1)	412 (97)
Cerebrovascular disease	404	12 (3)	8 (2)	384 (95)
Congestive heart failure	239	9 (4)	2 (1)	228 (95)
Myocardial infarction	84	5 (6)	1 (1)	78 (93)
E. Respiratory diseases	93	1 (1)	1 (1)	91 (98)
Asthma	73	1 (1)	1 (1)	71 (97)
F. Digestive diseases	328	20 (6)	8 (2)	300 (92)
Chronic liver disease	165	13 (8)	4 (2)	148 (90)
Peptic ulcer disease	31	-	2 (7)	29 (94)
G. Genitourinary disease	137	6 (4)	3 (2)	128 (93)
Chronic renal failure	112	5 (5)	2 (2)	105 (94)
**III. Injuries**	233	13 (6)*	15 (6)	205 (88)
A. Unintentional	102	2 (2)	6 (6)	94 (92)
Road traffic accidents	83	1 (1)	5 (6)	77 (93)
B. Intentional (Suicide…)	79	8 (10)*	7 (9)	64 (81)
Poisoning	28	2 (7)*	3 (11)	23 (82)
Undetermined	254	2 (1)*	4 (2)	248 (98)

Note: * P < 0.03. P value obtained by comparison of frequent khat use, occasional khat use and non khat use by chi- squared trend test.

**No *khat* use is a reference category.

### Association socioeconomic and behavioral risk factors with causes of death

Some demographic and socio-economic factors have shown some statistically significant associations with assigned causes of deaths (data were presented only for those associations that were significant). Accordingly, females were 1.96 and 1.89 times more likely to have died due to HIV/AIDS and malignancy respectively than males (Tables 6 and 7). Among different age groups, those within the age range of 25–44 years were 2.32 times more likely to have died due to HIV/AIDS compared to those with the age range of 15–24 years. On the other hand, those in the age group of 55 years and above were less likely to have died from HIV/AIDS compared to those in the age group of 15–24 years (Table 6). There were also significant associations between age and both cardiovascular and malignancy related causes of mortality. Those with age 45 years and above were 2 to 6 times more likely to have died due to cardiovascular diseases as compared to those with age 15–24 years. Likewise, those with age 55–64 years were 2.36 times more likely to have died from malignancy related causes as compared to those with age 15–24 years.

**Table 6:** Association of socioeconomic factors with HIV/AID caused mortality (N = 707), Addis Ababa, Ethiopia, 2006-2009

**Socioeconomic factors**	**HIV/AIDS**				
	**n (%)**	**COR ( 95% CI)**	**P**	**AOR ( 95% CI)**^**a**^	**P**
**Sex**					
M	314(9)	Ref.	NA	Ref.	NA
F	393(11)	1.34 (1.13-1.58)	0.001	1.96 (1.54-2.49)	<0.001
**Age**					
15-24	56 (2)	Ref	NA	Ref.	NA
25-44	468(13)	2.28 (1.64-3.16)	<0.001	2.32 (1.64-3.27)	<0.001
45-54	120(3)	0.94 (0.65-1.36)	0.742	0.88 (0.59-1.30)	0.515
55-64	43 (1)	0.29 (0.19-0.46)	<0.001	0.26 (0.16-0.42)	<0.001
≥65	20 (1)	0.02 (0.01-0.04)	<0.001	0.07 (0.04-0.13)	<0.001
**Education**					
< High School	439(12)	Ref	NA	Ref.	NA
≥ Secondary School	268(7)	1.77 (1.49-211)	<0.001	0.61(0.50-0.76)	<0.001
**Occupation**					
Manual Labor	535 (14)	Ref.	NA	Ref.	NA
Employed	953 (26)	0.67 (0.53-0.84)	0.001	0.99(0.75-1.29)	0.910
Unemployed	294 (8)	0.54 (0.39-0.75)	<0.001	0.86(0.59-1.26)	0.446
Housewives	1098(30)	0.24 (0.18-0.30)	<0.001	0.62(0.44-0.87)	0.005
Retired	544 (15)	0.05 (0.03-0.09)	<0.001	0.58(0.31-1.10)	0.094
Others	98 (3)	0.31 (0.17-0.57)	<0.001	0.30(0.16-0.57)	<0.001
Unknown	187 (5)	0.76 (0.53-1.10)	0.142	1.01(0.67-1.53)	0.952

Abbreviations: *COR* crude odd ratios, *AOR* adjusted odd ratios, *CI* confidence interval, *NA* not applicable.

^a^Adjusted for sex, age, religion, marital status, ethnicity, educational status, occupation.

**Table 7:** Association of socioeconomic factors with cardiovascular (N = 885) and malignancy (N = 386) caused mortality in Addis Ababa, Ethiopia, 2006-2009

**Socioeconomic factors**	**Cardiovascular diseases**				
	**No (%)**	**COR (95% CI)**	**P**	**AOR ( 95% CI)**^**a**^	**P**
**Age**					
15-24	19 (1)	Ref.	NA	Ref.	NA
25-44	77 (2)	0.82 (0.48-1.38)	0.45	0.74 (0.42-1.28)	0.276
45-54	95 (3)	2.53 (1.49-4.25)	<0.001	2.17 (1.22-3.86)	0.008
55-64	137 (4)	4.38 (2.63-7.30)	<0.001	3.98 (2.22-7.13)	<0.001
65-74	231 (6)	6.54 (3.97-10.77)	<0.001	5.97 (3.33-10.68)	<0.001
≥75	326 (9)	6.08 (3.72-9.92)	<0.001	5.84 (3.26-10.48)	<0.001
**Occupation**					
Manual Labor	535 (14)	Ref.	NA	Ref.	NA
Employed	953 (26)	2.30 (1.68-3.16)	<0.001	1.92(1.37-2.71)	<0.001
Unemployed	294 (8)	1.80 (1.19-2.70)	0.005	1.36 (0.88-2.11)	0.171
Housewives	1098 (30)	3.74 (2.76-5.07)	<0.001	1.55 (1.07-2.26)	0.021
Retired	544 (15)	4.86 (3.50-6.74)	<0.001	1.69 (1.17-2.42)	0.005
Others	98 (3)	1.31 (0.69-2.50)	0.415	2.31 (1.13-4.74)	0.022
Unknown	187 (5)	1.77 (1.10-2.83)	0.018	1.56 (0.95-2.61)	0.08
	**Malignancy**				
**Sex**					
Male	153 (4)	Ref	NA	Ref.	NA
Female	233 (6)	1.62 (1.30-2.00)	<0.001	1.89 (1.37-2.60)	<0.001
**Age**					
15-24	21 (1)	Ref	NA	Ref	NA
25-44	73 (2)	0.69 (0.42-1.15)	0.115	0.75 (0.43-1.30)	0.304
45-54	68 (2)	1.54 (0.92-2.58)	0.101	1.80 (1.01-3.26)	0.051
55-64	79 (2)	1.92 (1.15-3.20)	0.012	2.36 (1.28-4.35)	0.006
65-74	79 (2)	1.42 (0.85-2.24)	0.178	1.80 (0.96-3.37)	0.066
≥75	65 (1)	0.74 (0.44-1.24)	0.253	0.96 (0.50-1.83)	0.902
**Education**					
< High School	270 (7)	Ref	NA	Ref	NA
≥ Secondary School	116 (3)	1.12(0.89-1.41)	0.333	1.49 (1.12-1.99)	0.006
**Occupation**					
Manual labor	535 (14)	Ref.	NA	Ref.	NA
Employed	953 (26)	1.51 (1.01-2.26)	0.047	1.34 (0.88-2.04)	0.179
Unemployed	294 (8)	0.82 (0.45-1.51)	0.530	0.86 (0.46-1.60)	0.639
Housewives	1098 (30)	2.26 (1.54-3.32)	<0.001	1.72 (1.10-2.69)	0.018
Retired	544 (15)	1.77 (1.15-2.74)	0.010	1.81(1.11-2.94)	0.017
Others	98 (3)	1.99 (1.00-3.99)	0.052	2.32 (1.16-4.83)	0.024
Unknown	187 (5)	1.90 (1.09-3.34)	0.025	1.56 (0.87-2.78)	0.135

Abbreviations: *COR* crude odd ratios, *AOR* adjusted odd ratios, *CI* confidence interval, *NA* not applicable.

^a^Adjusted for sex, religion, marital status, ethnicity, educational status and occupation.

With regard to educational status, compared to other educational status categories, those with secondary school and above education were less likely to have died from HIV/AIDS (AOR = 0.61, p < 0.001), while they were more likely to have died from malignancy related causes (AOR = 1.49) compared to educational status of bellow secondary school. Housewives were less likely to have died from HIV/AIDS compared to manual laborers but more likely with cardiovascular diseases and malignancy. The employed were more likely to have died with cardiovascular diseases while the retired were from both cardiovascular and malignant diseases compared with manual laborers (Tables 6 and 7).

With regard to risk behaviors, as compared to non-alcohol consumers, those who were reported to have been frequent alcohol consumers were: three times more likely to have died due to chronic liver disease, twice more likely to have died due to HIV/AIDS, and twice more likely to have died due to tuberculosis. Furthermore, those who were reported to have been frequent tobacco smokers were twice more likely to have died due to tuberculosis (P = 0.010) as compared to those reported to be non-smokers (Table 8).

**Table 8:** Association between health risk behaviors with HIV/AIDS, tuberculosis, and chronic liver disease caused mortality in Addis Ababa, Ethiopia, 2006-2009

**Health risk behaviours**	**HIV/AIDS**				
	**No (%)**	**COR (95% CI)**	**P**	**AOR ( 95% CI)**^**a**^	**P**
**Alcohol use**					
Non alcohol use	506 (14)	Ref.	NA	Ref	NA
Frequent alcohol use	111 (3)	1.40 (1.12-1.78)	0.005	2.14 (1.52-3.00)	<0.001
Occasional alcohol use	90 (2.4)	0.84 (0.66-1.07)	0.162	1.21 (0.89-1.66)	0.231
	**Tuberculosis**				
**Alcohol use**					
Non alcohol use	299 (8)	Ref.	NA	Ref	NA
Frequent alcohol use	82 (2.2)	1.77 (1.35-2.31)	<0.001	1.61 (1.15-2.24)	0.005
Occasional alcohol use	79 (2.1)	1.33 (1.02-1.74)	0.035	1.35 (1.00-1.82)	0.056
**Tobacco use**					
Non tobacco use	395 (11)	Ref.	NA	Ref	NA
Frequent tobacco use	57 (2)	2.24 (1.64-3.07)	<0.001	1.67 (1.13-2.47)	0.010
Occasional tobacco use	8 (0.2)	2.93 (1.29-6.65)	0.010	2.41 (1.01-5.74)	0.047
	**Chronic liver diseases**				
**Alcohol use**					
Non alcohol use	92 (3)	Ref.	NA	Ref	NA
Frequent alcohol use	45 (1.2)	3.12 (2.15-4.52)	<0.001	3.09 (1.95-4.89)	<0.001
Occasional alcohol use	28 (1)	1.51 (1.00-2.32)	0.064	1.51 (0.94-2.42)	0.088

Abbreviations: *COR* crude odd ratios, *AOR* adjusted odd ratios, *CI* confidence interval, *NA* not applicable.

^a^Adjusted for sex, age, religion, marital status, ethnicity, educational status, occupation, alcohol use, tobacco use, and khat use.

## Discussion

Understanding the various risk factors for health is key to developing strategies for the prevention and control of diseases and injuries. A particular disease or injury is often caused by more than one risk factor, which means that multiple interventions are available to target each of these risks. In turn, most risk factors are associated with more than one disease, and targeting those factors can reduce multiple causes of disease [2]. In this regard, findings of this study are vital for health decision making and planning in Ethiopia and other countries with similar settings.

This study used burial surveillance with verbal autopsy approach to assess the association of socioeconomic and behavioral factors with causes of mortality in a country where vital registration system is not yet functioning. Although the verbal autopsy method has been recommended for decades in the settings of developing countries [17], the results of the present study have shown that the burial surveillance with verbal autopsy method is a feasible and sustainable approach from social, cultural and economic perspectives.

However, despite all efforts within the burial surveillance system to minimize it, the possibility of some level of bias including recall bias and accuracy of lay recall (knowledge of duration/temporality of consumption of substances) and the interaction between the risk behaviours and socioeconomic factors cannot be ruled out in this method. In addition, some individuals are also buried without the presence of close relatives or friends who could provide the verbal autopsy information and it is also possible that some residents may die or be buried outside the capital, just as non-residents of the city could be buried within the city limits [13].

In this study the prevalence of the three behavioural risk factors namely alcohol, tobacco and *khat* consumption among the deceased adults were measured. The measure used was the frequency of consumption of substances since it is difficult to measure the amount of substances consumed with the verbal autopsy method. On the other hand, such a measure can also serve the purpose, since the relationship between substance consumption and causes of death also depends on the frequency as well as the amount of consumption [1].

The analysis in this study has revealed frequent alcohol, tobacco and *khat* consumption as being more prevalent among the deceased males as compared to those of females in Addis Ababa. With regard to frequent alcohol consumption, it was reported among 22% of the males as compared to only 2% of females. This is in line with the reports of the Ethiopian Demographic and Health Survey (EDHS) where higher proportion (21%) of males in Addis Ababa reported to have ever drank alcohol as compared to females (12%) [23]. Almost all who were reported to have smoked tobacco in the present study were males. Similarly, the EDHS for Addis Ababa has reported 8% of males age 15–49 years as smoking tobacco with the percentage for females being negligible. The findings regarding *khat* chewing (where 8% of the males and 1% of the females among the deceased were reported to have frequently chewed *khat*) were also almost in line with the results of an earlier study in Addis Ababa that reported the behavior among 16% of males and 1% of females reported in Addis Ababa with higher proportions (16%) of males than females (1%) [19].

In this study, behavioral risk factors (frequent alcohol consumption and tobacco smoking) were also highly prevalent among the deceased individuals. Frequent *khat* chewing was also widely prevalent. This may indicate that these behavioral risk factors could have contributed to deaths due both to communicable and non-communicable causes, even though the level of contributions to each of the categories requires further study.

Associations of behavioral risk factors were identified among deaths due to cardiovascular diseases, HIV/AIDS, Tuberculosis, malignant neoplasm and chronic liver diseases, but not among deaths due to other causes. These specific causes of mortality were also reported as being the leading causes of death in the study area [13]. Moreover, HIV/AIDS was found to be associated with frequent alcohol consumption, a finding which is in line with previous studies that indicated a strong association of alcohol consumption with HIV incidence [24]. Tuberculosis was also found to be associated with both frequent alcohol and tobacco consumption, which is also supported by the existing evidences on the strong association between heavy alcohol consumption and tuberculosis [25]. The association between reported frequent alcohol consumption among those whose deaths were ascribed to chronic liver disease is also supported by the established direct relationship between higher levels of alcohol consumption and rising risk of some liver diseases [1].

With regard to demographic factors, the study has shown that females and those individuals in the age group of 25–44 years were more likely to have died from HIV/AIDS. This may be due to the fact that females are more susceptible to infectious diseases, and due to the fact that individuals in the age group 25–44 years are relatively more active sexually [2]. Furthermore, adults with age 45 years and above were found to be more likely to have died from cardiovascular diseases compared to those with the age 15–24 years. Similarly, individuals aged 55–64 years and females were more likely to have died due to malignancies. Among other reasons, these may be due to the emerging epidemiologic transition where the population is being exposed to the increasing risk of dying from cardiovascular and malignant diseases [19], even though the association observed between sex and malignancy requires to be further studied. Regarding educational status, those with secondary and higher education were less likely to have died from HIV/AIDS, and this is in line with an earlier report of the WHO [2].

## Conclusions

In spite of some of its limitations, the present study has shown frequent consumption of alcohol, tobacco and *khat* as being widely prevalent among deceased adults in Addis Ababa, particularly among males. These risk behaviors were also seen to be significantly more prevalent among those deceased due to HIV/AIDS, tuberculosis, malignancies, cardiovascular and chronic liver diseases. Moreover, being deceased due to HIV/AIDS and chronic liver diseases was positively associated with frequent alcohol consumption while being deceased due to tuberculosis was positively associated with both frequent alcohol and tobacco consumption. Being deceased due to HIV/AIDS was also positively associated with being female and with being in the age group of 25–44 years. On the other hand, being in the age group of 45 years and above was positively associated with being deceased from cardiovascular diseases, while being female, and being in the age group of 55–64 years is seen to be positively related with being deceased from malignancy.

Finally, it is recommended that any public health intervention targeting the leading causes of death (both communicable and non-communicable diseases) needs to address the behavioral risk factors of alcohol, tobacco and *khat* consumption. Further studies also should be conducted to analyze the contribution of these factors on the burden of mortality in Ethiopia and other developing countries.

## Competing interests

The authors declare that they have no competing interests.

## Authors' contributions

AM has made substantial contribution to the conception and design of the study; to the acquisition, analysis, and interpretation of the data; as well as to the preparation and revision of the manuscript. DHM has made substantial contribution to conception and design of the study; as well as to the preparation and revision of the manuscript. TA has made substantial contributions to conception and design of the study as well as to the preparation of the manuscript. All authors read and approved the final manuscript.

## Pre-publication history

The pre-publication history for this paper can be accessed here:

http://www.biomedcentral.com/1471-2458/13/634/prepub
